# What is the Role of Spatial Attention in Statistical Learning During Visual Search?

**DOI:** 10.5334/joc.382

**Published:** 2024-07-11

**Authors:** Aidai Golan, Aniruddha Ramgir, Dominique Lamy

**Affiliations:** 1 School of Psychological Sciences, Tel Aviv University, IL; 2 Sagol School of Neuroscience, Tel Aviv University, IL

**Keywords:** Spatial attention, statistical learning, distractor-location suppression, visual search

## Abstract

Our ability to learn the regularities embedded in our environment is a fundamental aspect of our cognitive system. Does such statistical learning depend on attention? Research on this topic is scarce and has yielded mixed findings. In this preregistered study, we examined the role of spatial attention in statistical learning, and specifically in learned distractor-location suppression. This phenomenon refers to the finding that during visual search, participants are better at ignoring a salient distractor at a high-probability location than at low-probability locations – a bias persisting long after the probability imbalance has ceased. Participants searched for a shape-singleton target and a color-singleton distractor was sometimes present. During the learning phase, the color-singleton distractor was more likely to appear in the high-probability location than in the low-probability locations. Crucially, we manipulated spatial attention by having the experimental group focus their attention on the target’s location in advance of the search display, using a 100%-informative spatial precue, while the control group was presented with a neutral, uninformative cue. During the subsequent test phase, the color-singleton distractor was equally likely to appear at any location and there were no cues. As expected, the results for the neutral-cue group replicated previous findings. Crucially, for the informative-cue group, interference from the distractor was minimal when attention was diverted from it (during learning) and no statistical learning was observed during test. Intertrial priming accounted for the small statistical-learning effect found during learning. These findings show that statistical learning in visual search requires attention.

Humans are remarkably good at picking up regularities in their environment. This form of “learning from the distributional properties of sensory input across time and space” (Frost et al., 2019) is referred to as *statistical learning* and drives a variety of cognitive functions from language acquisition to social cognition. Statistical learning has been observed for a wide range of stimuli, such as linguistic and non-linguistic auditory stimuli (e.g., Saffran et al., 1996; 1999) as well as visual stimuli presented in spatial or temporal sequences (e.g., Fiser & Aslin, 2002; Turk-Browne et al., 2005), and is an important building block of virtually all current theories of information processing (see Conway, 2020; Frost et al., 2019 for reviews). In the present study, we focus on the relation between statistical learning and attention.

Many studies have shown that statistical learning can influence where we allocate our attention. For instance, Zhao and colleagues (Zhao et al., 2013; Yu & Zhao, 2015) showed that a target was found faster within a stimulus stream containing temporal regularities than within a random, noisier stream, even though these regularities were not predictive of the target’s location, color or identity. The authors concluded that attention is spontaneously biased towards statistical regularities. Other studies showed that in visual search, observers learn to prioritize or deprioritize locations where a target or a distractor, respectively, appears more frequently than at other locations (Jiang et al., 2014; Jiang, 2018; Golan & Lamy, 2023; Wang & Theeuwes, 2018a; 2018b; 2018c; Ferrante et al., 2018; Britton & Anderson, 2020). These attentional biases were found to persist for several hundreds of trials after the regularity is discontinued (i.e., after the target or distractor becomes equally likely to appear in each location).

By contrast, relatively few studies have investigated the opposite relationship, that is, the extent to which attention modulates statistical learning. They manipulated different forms of attention and yielded inconsistent findings (Conway, 2020).

## The role of attention in statistical learning

Most of these studies examined the role of *feature-based attention*. Turk-Browne et al. (2005) presented their participants with two interleaved streams of shapes, each of a different color, and had them attend to just one color. A temporal regularity was embedded in one of the two colored streams. The authors found that participants learned the regularity only when it occurred in the stream with the relevant color. They concluded that statistical learning requires attention. However, this finding proved difficult to replicate (Musz et al., 2015; see also Hall et al., 2015 vs. Horvath et al., 2020, for additional conflicting findings).

Duncan and Theeuwes (2020) explored whether *task relevance* – rather than feature-based attention – influences statistical learning. They asked whether participants would learn to suppress the frequent location of a salient object when the task did not require search. Displays consisted of a circle among diamonds or vice-versa, with one of the non-unique shapes drawn in a unique color (red among green or vice-versa) on a subset of the trials. During learning, participants had to report the global configuration of the stimulus array (either a diamond or a circle). The authors reasoned that in this case, the regularity was not task relevant because none of the items forming the configuration had a target or distractor status, and they surmised that there was therefore no need to suppress the color singleton. They tested whether the regularity was nevertheless learned by having participants search for the unique shape in a subsequent test phase. The results showed that responses to the target were faster when the color singleton appeared in the high- than in low-probability locations (from the learning phase), suggesting that participants learned to suppress the high-probability location. The authors concluded that statistical learning could occur “in the absence of explicit top-down attention”.

Note that several aspects of Duncan and Theeuwes’ (2020) study mitigate this conclusion. First, the presence of the color singleton interfered with configuration judgments during the learning phase, and to a lesser extent when it appeared in the high- than in the low-probability location (Experiment 4). These findings invalidate the author’s critical assumption that ignoring the color singleton was task irrelevant. Second, the authors did not compare the magnitude of statistical learning when suppressing the color singleton was presumably task irrelevant relative to when it was task relevant. Thus, task relevance might still play an important role in statistical learning.^1^

Finally, a small number of studies investigated the influence of *spatial attention* on statistical learning. Baker et al. (2004) presented their participants with two shapes, one above the other, that were either connected by a bar or unconnected. Some shape combinations were more likely than others. The relevant finding for the present purposes is that when the shapes were unconnected, the regularity had an effect only when participants were instructed to attend to both locations (attended condition) but not when they had to attend to only one location (unattended condition). The authors concluded that statistical learning requires spatial attention. However, the interaction between the manipulation of the statistical regularity (i.e., the shape-combination frequency) and attention did not reach significance. In addition, Baker et al. (2004) did find a significant statistical-learning effect in the unattended condition in the first block of trials, after which performance improved dramatically – a finding suggesting that the absence of a learning effect in the unattended condition across the experiment may have resulted from a floor effect.

Failing and Theeuwes (2020) also reported findings that can shed light on the influence of spatial attention on statistical learning: they found that learned suppression of a high-probability distractor’s location increases with the salience of this distractor. As it is reasonable to assume that the more salient a distractor is, the more likely it is to receive spatial attention, this finding is consistent with the notion that statistical learning increases with spatial attention. However, Failing and Theeuwes’ (2020) study included only a learning phase, where high-probability location distractors were far more likely to appear at the same location on consecutive trials than low-probability location distractors. As a result, learned distractor-location suppression (i.e., statistical learning) was contaminated by intertrial priming. The authors addressed this issue by removing trials in which the distractor’s location had repeated from the previous trial. However, this procedure does not suffice to neutralize the effects of inter-trial priming, because the memory traces that underlie intertrial priming have been shown to exert their influence over several trials (e.g., Maljkovic & Nakayama, 1994; see also Golan & Lamy, 2023). A more effective procedure to ensure that statistical learning cannot be accounted for by intertrial priming is to use an extinction phase where the statistical regularity is no longer present (e.g., Golan & Lamy, 2022). In addition, Failing and Theeuwes (2020) did not report whether the distractor’s salience modulated intertrial priming – which is the most relevant finding to determine whether salience increased statistical learning or intertrial priming.

## Overview of the present study

As is clear from the foregoing review, whether attention influences statistical learning is, to date, an unresolved question. Here, we focused on spatial attention and investigated the extent to which it modulates learned distractor-location suppression.

**Figure 1:**
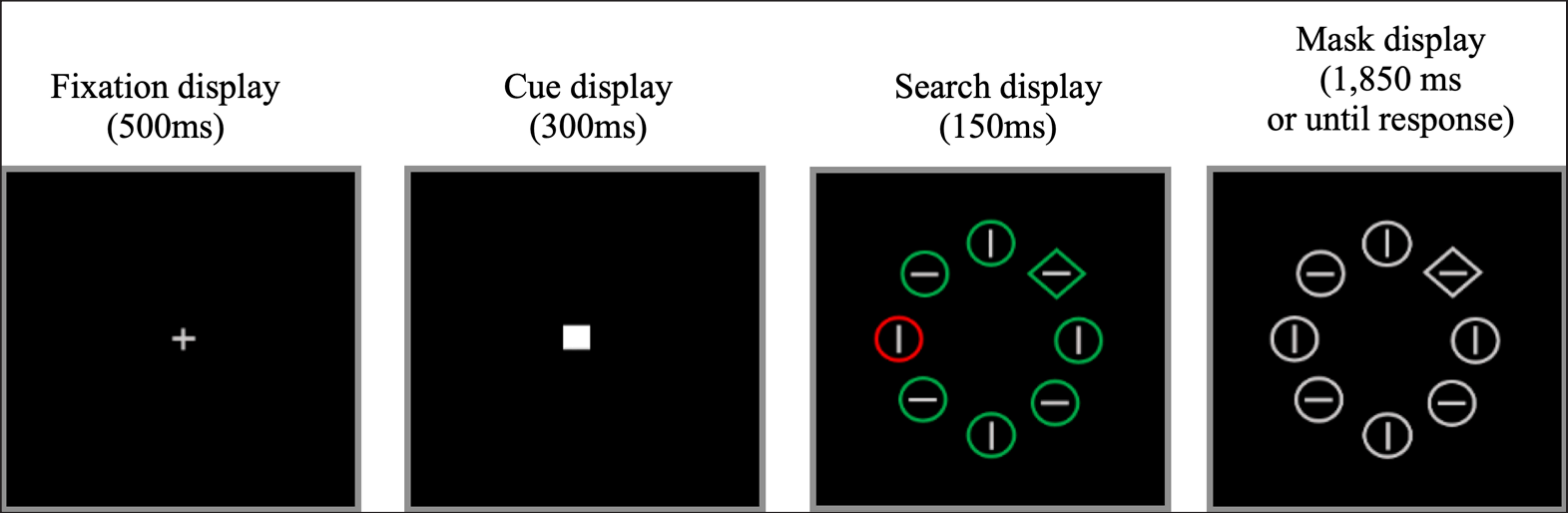
Sample displays during the *learning* phase of Experiment 1. Participants searched for the unique shape (either the unique diamond among circles or the unique circle among diamonds), while ignoring the color-singleton distractor, when present (2/3 of the trials). The search display was quickly followed by a white mask. During the *test* phase, the cue display was omitted. Not drawn to scale.

As in previous studies investigating this form of statistical learning (e.g., Wang & Theeuwes, 2018a; 2018b; 2018c; Ferrante et al., 2018), participants in the present study were instructed to search for a shape-singleton target and to ignore an irrelevant-color singleton (Figure 1). This color singleton, when present, appeared more often at one than at other locations during the learning phase. The subsequent test phase was similar, except that the color-singleton distractor was equally likely to appear at each location. The critical manipulation concerned the distribution of attention during the learning phase. For one group (the informative-cue group), a 100%-valid central arrow followed by a 100%-valid peripheral cue directed spatial attention to the target location prior to the search-display onset (Figure 3). Thus, we expected spatial attention to be tightly focused on the target’s location and the irrelevant-color singleton to receive little or no attention (e.g., Theeuwes, 1991; Yantis & Jonides, 1990). For the other group, the precue was uninformative (neutral-cue group) and the distractor was therefore expected to receive attention (e.g., Theeuwes, 1992). The colors of the search display were masked shortly after its onset, a procedure aimed at further reducing the probability that participants would shift their attention to the color-singleton distractor after locating the target in the informative-cue condition. We measured *distractor interference* as a performance cost on low-probability-distractor trials relative to distractor-absent trials and *statistical learning* as a performance benefit on high- vs. low-probability location trials.

The present design addressed the issues we had raised with regard to previous findings. By comparing statistical learning when a 100%-valid spatial cue diverted spatial attention away from the location where the statistical regularity occurred (informative-cue condition), relative to when spatial attention was distributed across the search display (neutral-cue condition), we unambiguously manipulated spatial attention. In addition, by using a learning-test design, we were able to measure statistical learning without contamination from inter-trial repetition effects: in the test phase, the distractor was equally likely to appear at each location and distractor-location repetition was therefore equally likely for the high- and for low-probability distractor locations. Finally, floor effects were not a concern in our study because the pre-registered, preliminary data indicated that distractor interference was large for the informative-cue group in the test phase: these preliminary data corroborated our conjecture that after becoming accustomed to finding the target without having to search for it, participants in the informative-cue group should find it at least as difficult as participants in the neutral-cue group to ignore the color-singleton distractor (the preliminary results are accessible on OSF).

### Pre-registered predictions

Our pre-registered predictions are presented in Table 1. If our manipulations were effective, (1) the neutral-cue group should show statistical learning in both the learning and test phases, as in previous studies (e.g., Britton & Anderson, 2020, Exp. 2; Duncan & Theeuwes, 2020, Exp.3; van Moorselaar & Theeuwes, 2021) and (2) the informative-cue group should show better overall performance and smaller distractor interference, if any, than the neutral-cue group during the learning phase (e.g., Theeuwes, 1991; Yantis & Jonides, 1990).

**Table 1:** Detailed description of the critical hypotheses tested in Experiment 2, their corresponding analysis plans, justifications for sensitivity, and theoretical implications.


QUESTION	HYPOTHESIS	ANALYSIS PLAN	RATIONALE FOR DECIDING THE SENSITIVITY OF THE TEST FOR CONFIRMING OR DISCONFIRMING THE HYPOTHESIS	SAMPLING PLAN	INTERPRETATION GIVEN DIFFERENT OUTCOMES	THEORY THAT COULD BE SHOWN WRONG BY THE OUTCOMES

Do the 100%-informative spatial pre-cues reduce *distractor interference*?	Hypothesis 1 H1: The 100%-informative spatial pre-cues reduce *distractor interference*. H0: The pre-cues do not reduce *distractor interference*.	A 2 × 2 ANOVA with *distractor interference* (distractor at a low-probability location vs. absent) as a within-subject factor and group (informative- vs. neutral-cue) as a between-subjects factor (on the learning-phase data). The effect of main interest is the interaction between the two factors.	Based on Experiment 1, we expect the size of *distractor interference* for the neutral-cue group to be *d* = 1.6. Previous studies (Theeuwes, 1991; Yantis & Jonides, 1990) showed that when spatial attention is tightly focused on the upcoming target location, a salient distractor does not interfere with search. Therefore, we evaluated the required sample to observe the critical interaction assuming a *distractor interference* effect of *d* = 0 for the informative-cue group.	A power analysis indicated that we would need a sample of 16 participants (i.e., 8 in each group) to detect the critical interaction. Our preliminary data in Experiment 2 revealed *distractor interference* of *d* = 1.6 for the neutral-cue group and *d* = .94 for the informative-cue group. A power analysis indicated that a full sample of 30 participants in each group would suffice to detect a reliable interaction.	Finding that *distractor interference* is significantly larger for the neutral- than for the informative-cue group, would support H1. Finding that this interaction is not significant would support H0.	Support for H1 would invalidate the claim that focused attention does not weaken attentional capture by salient irrelevant objects. Support for H0 would invalidate the claim that focused attention reduces – let alone prevents – attentional capture by salient irrelevant objects (Theeuwes, 1991; Yantis & Jonides, 1990)

Does *statistical learning* persist when the probability imbalance is discontinued?	Hypothesis 2 H1: *Statistical learning* persists into the test phase. H0: *Statistical learning* does not persist into the test phase.	A planned comparison (i.e., a paired t-test) between the high- and low-probability location conditions for the neutral-cue group (on the test-phase data).	We selected the size of the *statistical learning* effect to be *d* = .67 based on Experiment 1.	A power analysis indicated that we would need a sample of 16 participants to detect a *statistical learning* effect of this size.	Replication (see Experiment 1).	Not applicable (replication).

Is statistical learning modulated by spatial attention?	Hypothesis 3 H1: Statistical learning is modulated by spatial attention. H0: Statistical learning is independent of spatial attention.	A mixed 2 × 2 ANOVA with statistical learning (high- vs. low-probability location) as a within-subject factor and group (informative- vs. neutral-cue group) as a between-subjects factor (on the test-phase data). The effect of main interest is the interaction between the two factors.	We expect *statistical learning* of size *d* = .67 for the neutral-cue group based on Experiment 1. No study has measured statistical learning while using 100%-informative spatial cues. We evaluated the required sample to observe the critical interaction assuming a statistical learning effect of *d* = 0 for the informative-cue group.	Frequentist ANOVA: A power analysis indicated that we would need a sample size of 88 participants (i.e., 44 in each group) to detect the critical interaction. Bayesian ANOVA: A power analysis indicated that we would need 100 participants (i.e., 50 in each group) to observe *BF_10_* > 3 supporting the presence of the critical interaction.	Finding that *statistical learning* interacts with group during test (*p* < .05 and *BF_10_* > 3 for the interaction) would support H1. Finding *that statistical learning* does not interact with group during test (*p* > .05 and *BF_01_* > 3 for the interaction) would support H0.	Support for H1 would invalidate the claim that statistical learning is independent of spatial attention (Duncan & Theeuwes, 2020). Support for H0 would invalidate the claim that statistical learning is modulated by or contingent on spatial attention (Baker et al., 2004).

If statistical learning is modulated by spatial attention, is spatial attention *necessary* for statistical learning?	Hypothesis 4 H1: Spatial attention is necessary for statistical learning to occur. H0: Spatial attention is not necessary for statistical learning to occur.	*Exploratory analysis A* A planned comparison on *distractor interference* (distractor at a low-probability location vs. absent) in the learning phase and a planned comparison on *statistical learning* (high- vs. low-probability location) in the test phase, for the informative-cue group.	*Exploratory analysis A* Given that a significant *distractor-interference* of size *d* = .95 corresponded to a 20-ms effect (SD = 21.8 ms), in Experiment 1), we will consider that *distractor-interference* is negligible if it is non-significant or smaller than *d* = .5 (roughly corresponding to a 10-ms effect). We will consider that *statistical learning* did not occur during the test phase, if it is non-significant or smaller than *d =* .2.	*Exploratory analysis A* A power analysis indicated that we would need a sample of 27 participants to detect *distractor interference* of *d* = .5. A power analysis indicated that we would need a sample of 156 participants to detect a *statistical learning* effect of *d* = .2	*Exploratory analysis A* Finding no *statistical learning* in the test phase would support H1 (irrespective of the size of *distractor interference* in the learning phase). Finding *statistical learning* in the test phase and a negligible *distractor interference* in the learning phase would support H0.	Support for H1 would invalidate the claim that spatial attention is unnecessary for statistical learning (Duncan & Theeuwes, 2020). Support for H0 would invalidate the claim that spatial attention is necessary for statistical learning (Baker et al., 2004).

		*Exploratory analysis B* A linear regression analysis between *statistical learning* in the test phase and *distractor interference* in the learning phase, for the informative-cue group. The main effect of interest is the regression coefficient (β) and the intercept (α).	*Exploratory analysis B* We will conduct this analysis only if SL is modulated by spatial attention, and therefore expect a significant correlation between *distractor interference* in the learning phase and *statistical learning* in the test phase. We will consider that this prediction is confirmed if the regression coefficient is significant and larger than *β* = .4 (which is equivalent to a Pearson’s correlation coefficient of size, *r* = .4).	*Exploratory analysis B* A power analysis indicated that we would need a sample of 46 participants to detect a *β* = .4 for the correlation between *distractor interference* and *statistical learning*.	*Exploratory analysis B* Finding that the intercept is not significantly different from zero would support H1. Finding that the intercept is significantly larger than zero would support H0.	


The critical predictions concerned the comparison of statistical learning between the two groups during the test phase (Hypothesis 3 in Table 1). If statistical learning is independent of spatial attention, the two groups should show statistical learning of similar magnitude, even though the color-singleton distractor received more attention in the neutral- than in the informative-cue group. Conversely, if statistical learning is modulated by spatial attention, the informative-cue group should show poorer statistical learning than the neutral-cue group during the test phase. Our main objective was to test these two hypotheses against each other.

We further noted that if the latter hypothesis was confirmed, it could indicate either (a) that spatial attention is necessary for statistical learning or (b) that spatial attention enhances statistical learning but is not necessary for it to occur. Our lab’s resources did not allow us to test enough participants in order to disentangle these possibilities. However, we conducted planned exploratory analyses on the data from the informative-cue group, examining three possible outcomes that would provide suggestive evidence in favor of or against necessity (Hypothesis 4 in Table 1). Finding that statistical learning is not significant during the test phase would support the former (necessity) hypothesis (i.e., that the distractor did not receive enough attention in the learning phase to allow statistical learning). Conversely, finding that distractor interference is null during the learning phase and statistical learning is significant during the test phase would support the latter (no-necessity) hypothesis. Finally, finding that statistical learning is significant during the test phase, but distractor interference also is significant during the learning phase despite our best effort to divert participants’ spatial attention away from the distractor, would be compatible with both hypotheses (a) and (b) and disentangling these would require additional exploratory analyses described in Table 1 (Hypothesis 4).

## Experiment 1

The objective of Experiment 1 was to ensure that we could find statistical learning with the current setup in both the learning phase (e.g., Wang & Theeuwes, 2018a; Stilwell et al., 2019, Exp. 2; van Moorselaar & Theeuwes, 2021) and the test phase (e.g., Britton & Anderson, 2020, Exp.2; Duncan & Theeuwes, 2020, Exp. 3). Therefore, it included only a neutral-cue group.

### Methods

#### Sampling plan

We ran a power analysis based on the effect size of the statistical learning effect in the test phase (Cohen’s *d* = .615) reported by Duncan & Theeuwes (2020). This study was the closest to our study in stimuli and design and it is known that subtle changes in the display characteristics or duration of the learning phase affect statistical learning observed in the test phase (see e.g., Failing & Theeuwes, 2020). We estimated the power by simulating a dataset containing the effect of interest on reaction times (measured by Cohen’s *d*) and determined the minimum number of participants required to detect this effect with 80% power and alpha = .05 (see Brysbaert, 2019 for a detailed explanation of the method and the OSF materials for the script).

This analysis revealed that a sample size of 34 participants would suffice to detect statistical learning in the test phase with 80% power (detecting statistical learning in the learning phase with 80% power required only 7 participants, as statistical learning is typically much larger in the learning than in the test phase (e.g., *d* = 1.1 vs. *d* = .615, respectively, in Duncan & Theeuwes, 2020). To ensure that the high-probability distractor location was fully counterbalanced across the 4 possible locations (see methods), we invited 36 participants.^2^

#### Participants

Thirty-six participants (mean age = 24.8 years, SD = 2.6, four left-handed, fourteen females) were recruited through Prolific (Prolific.co) and were allowed to run on the experiment only if they met the following criteria: they were aged between 18–35, had normal or corrected-to-normal vision and normal color vision, were fluent in English, did not have dyslexia, dyspraxia or ADHD, and had an approval rate of at least 90% on their past participations.

#### Ethics information

The research was approved by Tel Aviv University Ethics Committee, 0000285-5, and complies with all national and international (e.g., Declaration of Helsinki) ethical regulations. Informed consent was obtained from all human participants. Participants received an average hourly compensation of £8–12 on Prolific.

#### Apparatus

The experiment was programmed on PsychoPy3 (Pierce et al., 2019) using the PsychoJS library and was hosted by Pavlovia (pavlovia.org). It was allowed to run only on desktops or on laptops and would exit automatically if a smartphone or tablet was detected. Participants were instructed not to exit full screen, but in case this occurred, the experiment was programmed to re-size itself to match the new window size. Responses were collected from the computer keyboard. The consistency of stimulus sizes across different display setups (screen resolution and screen size) was achieved using Li et al.’s (2020) credit card adjustment procedure: participants were asked to place their credit cards against a rectangle shown on the screen and to use the up and down arrow keys to resize the rectangle until it matched the size of the card.

#### Stimuli

The fixation display consisted of a white cross (0.7° × 0.7°) in the center of the screen. In the cue display, the fixation cross was replaced with a white filled square (0.7° × 0.7°). In the search display (see Figure 1), eight shapes (drawn with a 0.01° stroke) were added, with their centers placed equidistantly on an imaginary circle (2.6° in radius). The target was the unique shape among homogeneously shaped non-targets, either the unique circle among diamonds or vice-versa (1.6° in diameter for circles and 1.4° in side for diamonds). Each shape contained a gray line (0.5° in length), either horizontal or vertical, in its center. On distractor-absent trials, the search items were either all green or all red. On distractor-present trials, one of the non-targets (the color-singleton distractor) appeared in a different color: either red among green or green among red. The mask display was similar to the search display, except that all shapes became gray, thereby obliterating the information necessary to locate the color-singleton distractor. Color coordinates were red: RGB (255, 0, 0), green: RGB (0, 255, 0) and gray: RGB (88, 88, 88). All stimuli were presented against a black background.

#### Procedure and design

The experiment included a practice phase, a learning phase, and a test phase (in this order). Trials in the practice phase were similar to the trials in the learning phase. In both the learning and test phases, the color-singleton distractor was present on 2/3 of the trials and absent on 1/3 of the trials. The participants’ task was to report the orientation of the line inside the target by pressing the “m” or the “k” key when the line was horizontal or vertical, respectively, with their right hands on the keyboard, as fast and as accurately as possible. The target was equally likely to be a circle among diamonds or a diamond among circles. It was equally likely to appear at each possible location and its location never overlapped with the distractor’s location. The line orientation inside each shape was randomly determined, with the constraint that each search display contained exactly four horizontal and four vertical lines.

##### Learning phase

Each trial sequence consisted of the fixation display (500 ms), a spatially uninformative cue display (300 ms), a search display (150 ms), and the mask display (until response or 1,850 ms). A new trial began after a 500-ms blank inter-trial interval. An incorrect response or a time-out was followed by an error beep (225 Hz). Eye movements were monitored but participants were explicitly instructed to maintain fixation.

The color-singleton distractor, when present, appeared in one location on 65% of the trials (high-probability location) and in each of the other seven locations on 5% of the trials (low-probability locations). For each participant, the high-probability location was one of the four cardinal locations (top, bottom, left or right) and remained the same throughout the learning phase. Which location served as the high-probability location was counterbalanced across participants.

##### Test phase

The test phase was similar to the learning phase except for the following changes. The cue display was omitted from the sequence of events. The color-singleton distractor was equally likely to appear in each of the eight possible locations.

There were 30 practice trials. The learning phase included 360 trials divided into three 120-trial blocks and the test phase included 240 trials divided into two 120-trials blocks. Participants were allowed a short self-paced break of a maximum of three minutes after each block of trials. All within-subject conditions were randomly intermixed within each block of trials.

#### Exclusion criteria

In both experiments, responses faster than 200 ms (anticipatory responses) were excluded from all RT and accuracy analyses. Any participant who responded on fewer than 90% of the trials or performed with less than 70% accuracy was automatically replaced with a new participant. Further, any participant with either an average RT or an average accuracy rate differing from the group’s mean by more than 3 standard deviations was replaced. Outlier-RT trials (defined as trials with an RT exceeding their cell’s mean by more than 2.5 SDs) as well as error trials and timeouts were excluded from all RT analyses.

Whenever the sphericity assumption was violated for the ANOVAs, we reported the *p*-values after correcting them with the Greenhouse-Geisser procedure.

### Results

**Figure 2:**
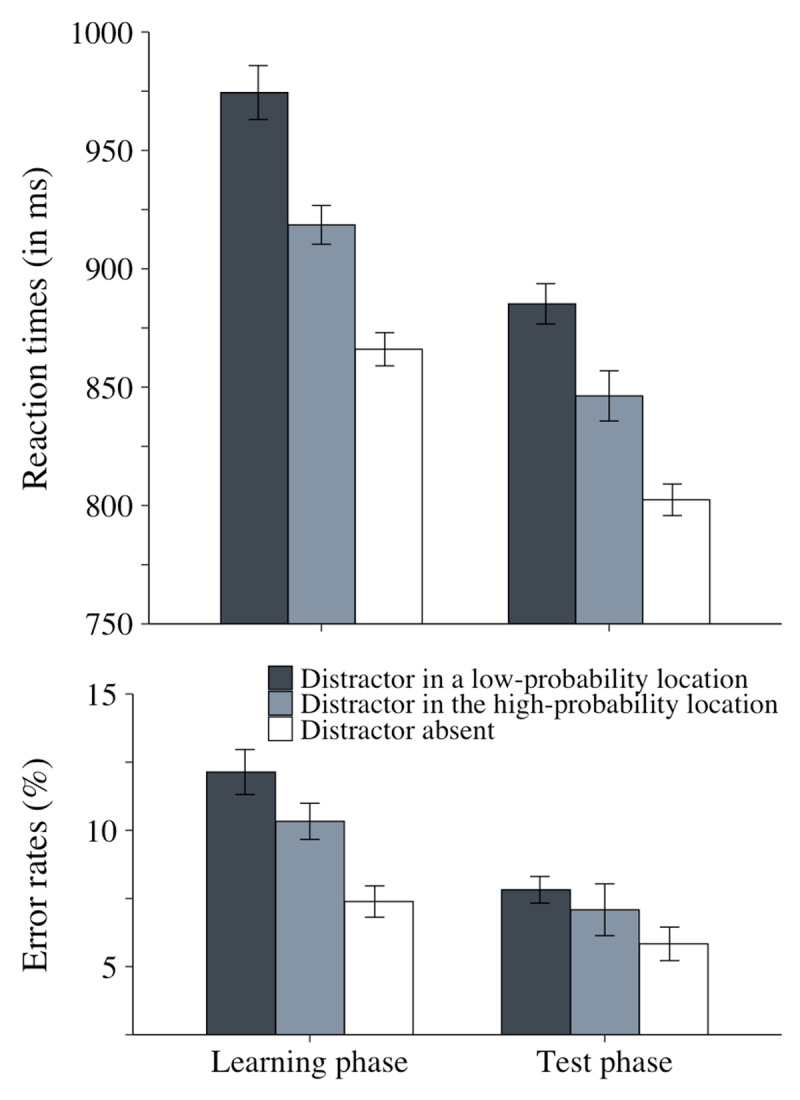
Mean RTs (in millseconds) and mean % of errors as a function of distractor condition (present at a low-probability location, present at the high-probability location, or absent) in the learning and test phases in Experiment 1. The error bars represent within-subject standard error of the mean.

No participant met any of the exclusion criteria. Error trials (1.6% in the learning phase and 1% in the test phase) as well as RT-outlier trials (2.2% of the remaining trials in the learning phase and 2.8% in the test phase) were excluded from the RT analyses. The RT and accuracy results are plotted in Figure 2.

#### Learning phase

We conducted an ANOVA on the learning phase data with distractor condition (absent, high-probability location, low-probability location) as a within-subject factor.

##### Reaction times

The main effect of distractor condition was significant, *F*(2, 70) = 66.4, *p* < .001, \[\eta _p^2\] = .65. Planned comparisons showed that both distractor interference, 108 ms, *t*(35) = 9.8, *p* < .001, *d* = 1.6, and statistical learning, 56 ms, *t*(35) = 5.4, *p* < .001, *d* = .9, were significant.

##### Accuracy

The accuracy data closely mirrored the RT data. The main effect of distractor condition was significant, *F*(2, 70) = 12.9, *p* < .001, \[\eta _p^2\] = .3. Planned comparisons revealed that both distractor interference, 4.7%, *t*(35) = 4.3, p = .001, *d* = .7, and statistical learning, 1.8%, *t*(35) = 2, *p* = .047, *d* = .3, were significant.

#### Intertrial priming in the learning phase

For comparability with Experiment 2, we conducted exploratory analyses on high-probability distractor-location trials, with distractor-location repetition (distractor on the previous trial also at the high-probability location vs. at a low-probability location) as a within-subject factor.

##### Reaction times

Search was significantly faster when the location of the distractor repeated than when it changed, 931 ms vs. 908 ms, respectively, *t*(55) = 3.27, *p* < .001, *d* = .55, that is, there was a 23-ms intertrial priming effect.

##### Accuracy

There were significantly fewer errors when the location of the distractor repeated than when it changed, 11.45% vs. 9.43%, respectively, *t*(55) = 1.98, *p* < .05, *d* = .33, that is, a 2.02% intertrial priming effect.

#### Test phase

We conducted an ANOVA on the test-phase data with distractor condition (absent, high-probability location, low-probability location) as a within-subject factor.

##### Reaction times

The main effect of distractor condition was significant, *F*(2, 70) = 45.7, *p* < .001, \[\eta _p^2\] = .56. Planned comparisons showed that both distractor interference, 83 ms, *t*(35) = 11.74, *p* < .001, *d* = 1.96, and statistical learning, 39 ms, *t*(35) = 4.04, *p* < .001, *d* = .67, were significant.

##### Accuracy

The main effect of distractor condition was not significant, *F*(2, 70) = 2.11, *p* =.13, \[\eta _p^2\] = .06.

## Experiment 2

The results of Experiment 1 confirmed that for the neutral-cue group, we were able to replicate the well-established statistical-learning effect known as the learned distractor-location suppression in both the learning phase (e.g., Wang & Theeuwes, 2018a; Stilwell et al., 2019, Exp. 2; van Moorselaar & Theeuwes, 2021) and the test phase (e.g., Britton & Anderson, 2020, Exp. 2; Duncan & Theeuwes, 2020, Exp. 3). In Experiment 2, we turned to testing our main research question: does spatial attention modulate statistical learning? Experiment 2 was pre-registered.

### Methods

#### Sampling plan and participants

We ran several a priori power analyses to determine our sample size (see Table 1). These analyses indicated that a sample size of 100 participants (50 participants in each group) was required to detect the weakest effect of interest in the current study (Hypothesis 3). To ensure that the high-probability distractor location was fully counterbalanced across the 8 possible locations,^3^ we invited 112 participants (56 in each group; mean age = 24.9 years, SD = 2.84 years, 106 right-handed, 63 females). They were recruited through Prolific (Prolific.co) following the same procedure as in Experiment 1.

#### Apparatus

The apparatus was similar to Experiment 1 except for the following changes. We added Li et al.’s (2020) blind-spot measurement procedure to estimate individual viewing distances and adjust stimulus sizes accordingly. We also added a color flicker-fusion procedure to ensure that the display colors, red and green, were equiluminant (e.g., Anstis and Cavanagh, 1983).

#### Stimuli, procedure, and design

**Figure 3:**
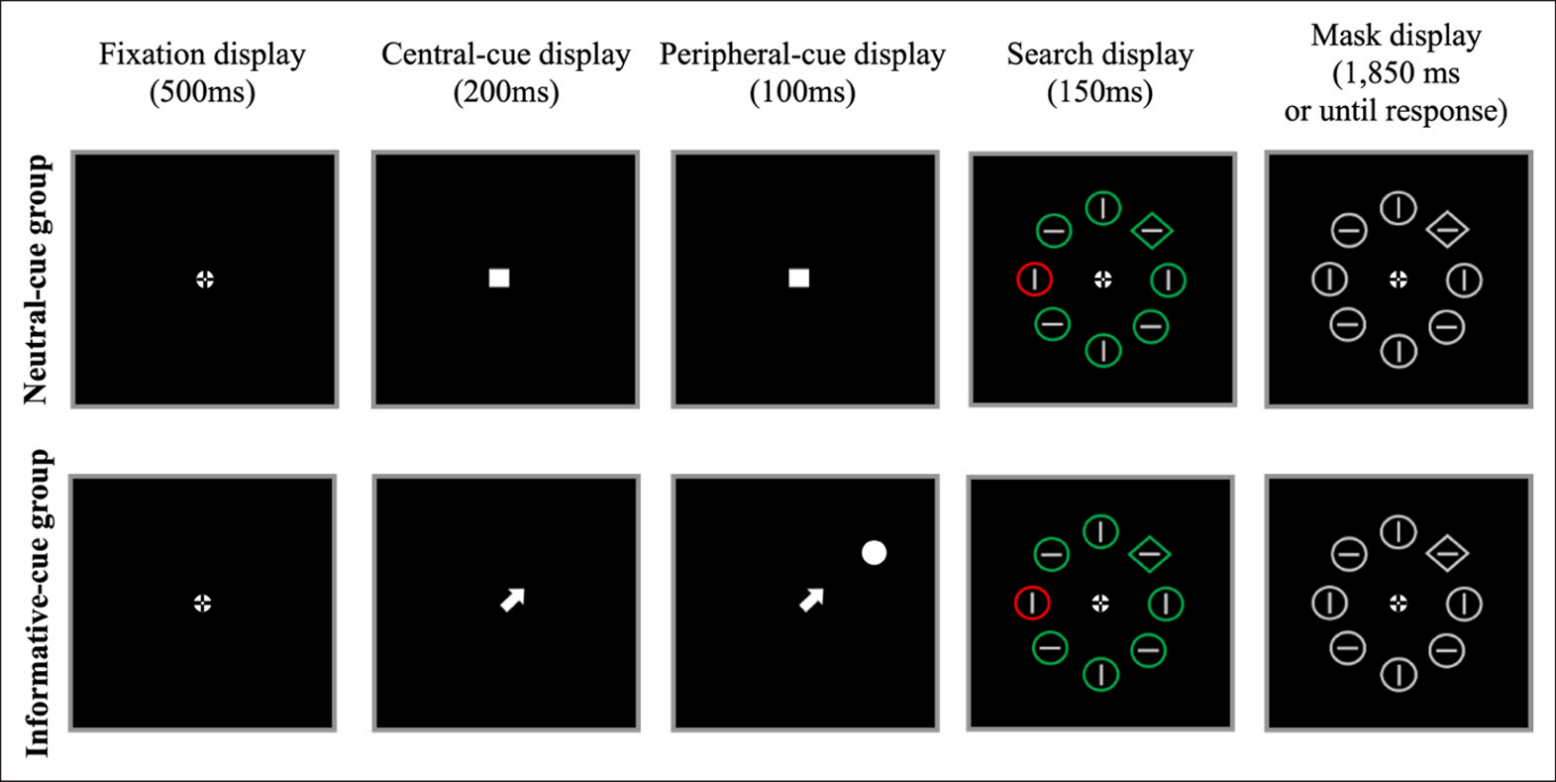
Sample displays during the *learning* phase of Experiment 2. As in Experiment 1, participants searched for the unique shape (here, the diamond), while ignoring the color-singleton distractor, when present (2/3 of the trials). For the informative-cue group (upper panel), the central arrow cue as well as the peripheral cue indicated the upcoming target’s location with 100% validity. During the *test* phase, the cue displays were omitted (the test phase was therefore be the same for the neutral- and informative-cue groups). Not drawn to scale.

The stimuli, procedure, and design were similar to those of Experiment 1, except for the following changes (see Figure 3). The fixation cross was replaced with a “bull’s eye” fixation cross (0.7° × 0.7°), which was shown to reduce involuntary eye-movements and promote stable fixations compared to other fixation signs (Thaler et al., 2013). This bull’s eye fixation cross appeared in the fixation, search and mask displays. The first-cue display was the same as the cue display in Experiment 1 (a white filled square) for the neutral-cue group and consisted of a white arrow (0.5° × 0.1° for the stem, 0.5° width of the head) for the informative-cue group. The second-cue display was similar to the central-cue display for both groups, except that for the informative-cue group, a white filled circle (0.7° in diameter) was added at an eccentricity of 2.8°. In the search display, the shapes was placed at an eccentricity of 2.8°. The color coordinates for green ranged from RGB (0, 128, 0) to RGB (0, 252, 0), adjusted for each participant with the flicker-fusion procedure to ensure that it was equiluminant with the red color.

The trial sequence in the learning phase included the fixation display (500 ms), the first cue display (200 ms), the second cue display (100 ms), a search display (150 ms), and the mask display (until response or 1850 ms). The trial sequence in the test phase was the same as in Experiment 1 for both groups. Prior to the practice session described in Experiment 1 (30 trials), all participants underwent a target-only practice (30 trials), in which the search display included only the target (and no distractors).

For each participant, the high-probability location was selected from the eight possible locations (and not only from the cardinal locations as was the case in Experiment 1). For the informative-cue group, both the arrow cue and the peripheral cue indicated the location of the upcoming target with 100% validity.

### Results

**Figure 4:**
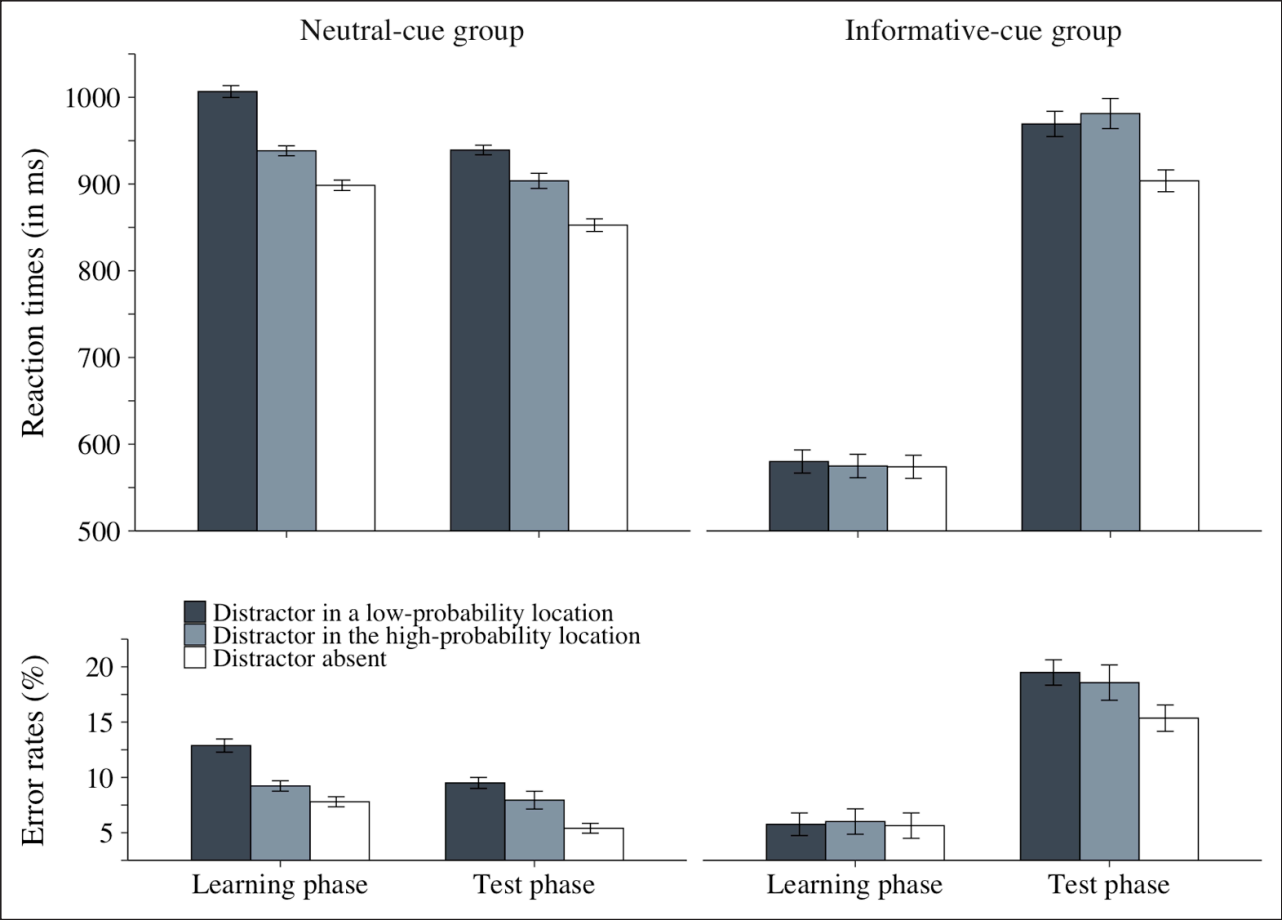
Mean RTs (in millseconds) and mean % of errors as a function of distractor condition (present at a low-probability location, present at the high-probability location, or absent) in the learning phase (left panel) and in the test phase (right panel), separately for the informative-cue and for the neutral-cue groups in Experiment 2. The error bars represent within-subject standard error of the mean.

Two participants from the neutral-cue group were replaced with new participants because they were accuracy outliers (mean accuracy = 71.9% and 74.4% vs. group’s mean = 91.2%). Error trials in the learning phase (9.97% and 5.80% for the neutral- and informative-cue groups, respectively) and in the test phase (7.62%, and 17.8% for the neutral- and informative-cue groups, respectively), as well as RT-outlier trials in the learning phase (1.44% and 1.78% for the neutral- and informative-cue groups, respectively) and in the test phase (.98% and 1.06% for the neutral- and informative-cue group, respectively) were excluded from all RT analyses. The RT and accuracy results are plotted in Figure 4.^4^ As planned in the pre-registration (Table 1), we reported Bayes factors for the results testing Hypotheses 3 and 4 (calculated on JASP with default priors; JASP Team, 2024).

#### Distractor interference in the learning phase

To verify that our manipulation of spatial attention was effective, we assessed its impact on distractor interference by conducting an ANOVA with distractor condition (distractor in a low-probability location vs. absent) as a within-subject factor and group (informative vs. neutral cue) as a between-subjects factor on the learning-phase data (Hypothesis 1).

##### Reaction times

Unsurprisingly, participants were faster in the informative- than in the neutral-cue group, *F*(1, 110) = 248.02, *p* < .001, \[\eta _p^2\] = .69. The main effect of distractor condition was also significant *F*(1, 110) = 206.79, *p* < .001, \[\eta _p^2\] = .65, but as predicted, it interacted with group, *F*(1, 110) = 164.76, *p* < .001, \[\eta _p^2\] = .60: distractor interference was considerably larger in the neutral-cue than in the informative-cue group, 108 ms vs. 6.14 ms, respectively, but was significant for both groups, *t*(55) = 14.30, *p* < .001, *d* = 1.91 and *t*(55) = 2.51, *p* < .01, *d* = .37, respectively.

##### Accuracy

The accuracy results closed mirrored the RT results. The main effects of group and distractor condition were both significant, *F*(1, 110) = 22.20, *p* < .001, \[\eta _p^2\] = .17 and *F*(1, 110) = 37.45, *p* < .001, \[\eta _p^2\] = .25, respectively. The two factors interacted, *F*(1, 110) = 34.04, *p* < .001, \[\eta _p^2\] = .24, indicating that distractor interference was significant for the neutral-cue group, 5.09%, *t*(55) = 7.35, *p* < .001, *d* = .98, and non-significant for the informative-cue group, .12%, *t*(55) = .25, *p* = .40, *d* = .03.

#### Distractor interference in the test phase

To evaluate the extent to which the singleton distractor interfered with search in the two groups during test, we conducted the same ANOVA on the test-phase data.

##### Reaction times

The main effect of distractor condition was significant, *F*(1, 110) = 165.19, *p* < .001, \[\eta _p^2\] = .60, indicating that search performance was slower when the color-singleton distractor was present in a low-probability location than when it was absent. The two-way interaction between distractor condition and group did not reach significance, *F*(1, 110) = 3.16, *p* = .08, \[\eta _p^2\] = .03, indicating that, unlike in the learning phase, the distractor interfered to a similar extent during test in the informative- and in the neutral-cue groups, 65.6 ms and 86.6 ms, respectively. The main effect of group was not significant, *F*(1, 110) = 1.68, *p* = .20, \[\eta _p^2\] = .02.

##### Accuracy

The accuracy findings did not show any speed-accuracy trade-off. The main effects of both distractor condition and group were significant, *F*(1, 110) = 60.79, *p* < .001, \[\eta _p^2\] = .36 and *F*(1, 110) = 22.57, *p* < .001, \[\eta _p^2\] = .17, respectively, with more errors in the informative-cue than in the neutral-cue group, 17.42% vs. 7.45%, respectively, and when the color-singleton distractor was present in a low-probability location than when it was absent, 14.5% vs. 10.4%, respectively. The interaction between the two factors was not significant, *F* < 1, indicating that during test, the distractor interfered to the same extent in the informative- and in the neutral-cue groups, 4.13% and 4.10%, respectively.

#### Statistical learning in the learning phase

To evaluate statistical learning during the learning phase, we conducted an ANOVA with distractor condition (distractor in the high-probability vs. in a low-probability location) as a within-subject factor and group (informative vs. neutral cue) as a between-subjects factor on the learning-phase data.

##### Reaction times

The main effects of group and distractor condition were significant, *F*(1, 110) = 270.16, *p* < .001, \[\eta _p^2\] = 71 and *F*(1, 110) = 86.64, *p* < .001, \[\eta _p^2\] = .44, respectively, and the interaction between the two factors was also significant, *F*(1, 110) = 63.85, *p* < .001, \[\eta _p^2\] = .38: statistical learning was considerably larger in the neutral-cue than in the informative-cue group, 68.3 ms vs. 5.20 ms, respectively, but was significant for both groups, *t*(55) = 9.22, *p* < .001, *d* = 1.23, and *t*(55) = 1.91, *p* = .03, *d* = .26, respectively.

##### Accuracy

The accuracy findings mirrored the RT findings. The main effects of group and distractor condition were both significant, *F*(1, 110) = 27.68, *p* < .001, \[\eta _p^2\] = 20 and *F*(1, 110) = 21.44, *p* < .001, \[\eta _p^2\] = .16, respectively. The two factors interacted, *F*(1, 110) = 28.16, *p* < .001, \[\eta _p^2\] = .20, indicating that statistical learning was significant for the neutral-cue group, 3.66%, *t*(55) = 5.87, *p* < .001, *d* = .78, but not for the informative-cue group, –.25%, *t*(55) = .63, *p* = .74, *d* = –.09.

#### Intertrial priming in the learning phase

Statistical learning in the learning phase is confounded with intertrial priming: high-probability location distractors are far more likely to appear at the same location on consecutive trials than low-probability location distractors. To determine the contribution of intertrial priming in the small statistical-learning effect found in the informative-cue group, we conducted an exploratory analysis to measure intertrial priming that is not contaminated by statistical learning. To do that, we conducted an exploratory ANOVA on high-probability distractor location trials, with distractor-location repetition (distractor on the previous trial also at the high-probability location vs. at a low-probability location) as a within-subject factor and group as a between-subjects factor.

##### Reaction times

The interaction between distractor-location repetition and group was significant, *F*(1, 110) = 16.00, *p* < .001, \[\eta _p^2\] = .13, indicating that inter-trial priming was larger in the neutral-cue than in the informative-cue group, 31.3 ms vs. 4.96 ms, respectively. Critically, however, intertrial priming was significant not only for the neutral-cue group, *t*(55) = 4.99, *p* < .001, *d* = .67 but also for the informative-cue group, *t*(55) = 2.47, *p* < .01, *d* = .33. Notably, this intertrial-priming effect was in the same order of magnitude as the statistical learning effect for the informative-cue group (4.96 ms vs. 5.20 ms, respectively).^5^

##### Accuracy

The interaction was not significant, *F* < 1.

#### Statistical learning in the test phase

To test our main predictions (Hypotheses 3–4), we conducted and ANOVA with distractor condition (distractor in the high-probability vs. in a low-probability location) as a within-subject factor and group (informative vs. neutral cue) as a between-subjects factor on the test-phase data.

##### Reaction times

Neither the main effect of group nor the main effect of distractor condition was significant, *F*(1, 110) = 2.61, *p* = .11, \[\eta _p^2\] = .02 and *F*(1, 110) = 3.03, *p* = .085, \[\eta _p^2\] = .03, respectively. Crucially, however, the two-way interaction between these factors was significant, *F*(1, 110) = 12.31, *p* < .001, \[\eta _p^2\] = .10, *BF_10_* = 52.38. Planned comparisons showed that statistical learning was significant for the neutral-cue group, 35.5 ms, *t*(55) = 4.01, *p* < .001, *d* = .54, *BF_10_* = 253.87, but not for the informative-cue group, –12 ms, *t*(55) = 1.17, *p* = .88, *d* = –.16, *BF_01_* = 13.93. In other words, we found clear evidence supporting our pre-registered Hypotheses 3 and 4.

To examine the possibility that in the informative-cue group statistical learning may have occurred but dissipated quickly, we conducted an exploratory analysis on the data of this group with block as an additional variable. We found no interaction between statistical learning and block, *F* < 1, and no statistical learning effect in either the first or the second block, *p* = .72, *d* = –.08, and *p* = .78, *d* = –.11, respectively .

##### Accuracy

There was no indication of a speed-accuracy trade-off. The main effect of group was significant, *F*(1, 110) = 20.63, *p* < .001, \[\eta _p^2\] = .16, indicating that there were more errors in the informative-cue than in the neutral-cue group, 19.03% vs. 8.72%, respectively. Neither the main effect of distractor condition nor the interaction between the two factors was significant, *F*(1, 110) = 2.00, *p* = .16, \[\eta _p^2\] = .02 and *F* < 1, respectively.

#### Impact of statistical learning on target processing

As a further test of whether the high-probability distractor location was suppressed, we examined whether search on distractor-absent trials was impaired when the target appeared at that location than at low-probability distractor locations (e.g., Britton & Anderson, 2020, Experiment 2). To do that, we conducted an exploratory ANOVA on distractor-absent trials of the test phase with target location (target at the high- vs. a low-probability distractor location) as a within-subject factor and group (informative vs. neutral cue) as a between-subjects factor.

##### Reaction times

Mirroring the analyses pertaining to statistical learning on distractor-present trials, the interaction between the target location and group was significant, *F*(1, 110) = 5.69, *p* < .05, \[\eta _p^2\] = .05, indicating that there was a significant performance cost when the target appeared at the high- than at a low-probability distractor location for the neutral-cue group, 34.5 ms, *t*(55) = 3.23, *p* < .01, *d* = .43, but not for the informative-cue group, –8.06 ms, *t*(55) =.56, *p* = .71, *d* = –.08.

##### Accuracy

The interaction between target location and group was not significant, *F* < 1.

## General discussion

The objective of the present study was to examine the influence of spatial attention on statistical learning. We examined this question by focusing on learned distractor-location suppression, a form of statistical learning by which observers learn to suppress the location where a salient distractor is most likely to occur. We investigated whether such learned suppression depends on how much spatial attention the salient distractor receives. Our findings provided a clear answer to this question: learned distractor suppression is strongly modulated by spatial attention and we found convincing evidence that it actually requires spatial attention.

### Summary of the findings

We first ensured that we could replicate learned distractor-location suppression with our experimental set-up (Experiment 1). As in previous studies, we found that when participants searched for a shape singleton, a color-singleton distractor interfered less when it appeared at the high-probability location than when it appeared at low-probability locations. This statistical-learning effect occurred both during learning (e.g., Wang & Theeuwes, 2018a), where the probability imbalance prevailed, and during test, where the probability imbalance was discontinued (e.g., Ferrante et al., 2018).

We addressed our critical question in Experiment 2: we compared statistical learning as well as distractor interference at the low-probability distractor locations, as a function of how much attention the distractor received during learning. For one group, attention was diverted away from the color-singleton distractor prior to each trial during learning (informative-cue group) and for the other, attention was unconstrained (neutral-cue group). During the test phase, for both groups, the cue as well as the distractor-location probability imbalance were removed. The findings for the neutral-cue group replicated the well-established pattern observed in Experiment 1. For the informative-cue group, during learning, we found virtually no distractor interference (6 ms vs. 108 ms in the neutral-cue group), confirming that our attention manipulation was effective. We also found a small statistical-learning effect (5 ms vs. 68 ms in the neutral-cue group), but it was entirely accounted for by intertrial priming (i.e., repetition of the distractor’s location on successive trials).

Our critical finding is that statistical learning did not occur for the informative-cue group: the large distractor interference found in the test phase was similar irrespective of whether the distractor appeared at the high- or a low-probability distractor location. By constrast in the neutral-cue group, interference was reduced by more than 40%. Taken together, these findings fully support our pre-registered hypotheses. Corroborating this pattern of results in an exploratory analysis, we also found poorer performance on distractor-absent trials when the target appeared at the high- than at a low-probability distractor location, only for the neutral-cue group and not for the informative-cue group.

Taken together, these findings support the idea that spatial attention is required for learned distractor suppression.

### Corollary findings

Two additional findings not directly related to our research question are worth noting. First, we found evidence for a performance benefit when the salient distractor appeared at the same location on successive trials in the neutral-cue group. Previous studies that examined this effect yielded mixed findings: Failing and Theeuwes (2020) reported a very large effect (with a Cohen’s *d* ranging from 1.6 to 1.8), whereas Wang and Theeuwes (2018a, *SP*) reported a small effect that did not reach significance. The likely reason for this difference is that Failing and Theeuwes (2020) did not disentangle the repetition effect from statistical learning: since they calculated the repetition effect across high-and low-probability distractor-location trials (with the former being faster than the latter) and since there was a larger proportion of high- than of low-probability distractor-location trials in the repetition condition than in the no-repetition condition, the effect of distractor-location repetition was contaminated by statistical learning. By contrast, as in the present study, Wang and Theeuwes restricted their analysis of distractor-location repetition to high-probability distractor-location trials, thereby circumventing contamination of the effect by statistical learning. It is likely that Wang and Theeuwes’ study (24 participants) was underpowered to detect the effect, because with a very similar design but larger samples (36 participants in Exp.1 and 56 in Exp.2), we found a reliable effect of distractor-location repetition in both our experiments.

The second cororally finding is that we provided novel evidence for the conclusion that salient events outside the focus of attention can be entirely ignored. Previous studies showed that an abruptly onset item that appears before the search array is presented can be successfully ignored when attention is focused on the upcoming target location in advance (Theeuwes, 1991; Yantis & Jonides, 1990). We generalized this findings to distractors that are salient by their unique color and appear simultaneously with the search array. One may claim that although distractor interference was very small in the informative-cue group (6 ms) it was nonetheless significant and that this finding might indicate that a distractor outside the focus of attention cannot be entirely ignored. We do not favor this interpretation. Considering that distractor interference in the neutral-cue group was much larger (108 ms), we find it more likely that some participants failed to use the cue on a small minority of trials, and that the small distractor interference we found resulted from these trials.

### Link to previous studies on the role of attention in statistical learning

While the current findings are consistent with the results from previous studies that examined the role of spatial attention in statistical learning (Baker et al., 2004; Failing & Theeuwes, 2020), they put the conclusion that statistical learning requires spatial attention on much firmer ground. Indeed, our study is the first to demonstrate that spatial attention is required for statistical learning while manipulating spatial attention to the stimulus to which the statistical regularity pertains, in a well-powered experiment, with an independent measure of the attentional manipulation, and with a design that invalidates alternative accounts to statistical learning (i.e., with a learning-test design).

However, further research is needed in order to determine whether our findings can be generalized from learned distractor suppression to other statistical-learning phenomena and from spatial attention to other forms of attention, such as feature-based attention (e.g., Turk-browne et al., 2005) and task relevance (Duncan & Theeuwes, 2020).

### Conclusions and limitations

While we replicated previous work by showing that learned distractor-location suppression is robust and long-lasting (e.g., Ferrante et al., 2018), we uncovered an important characteristic of this statistical-learning phenomenon: it is contingent on spatial attention. In other words, repeated exposure to a potentially distracting salient object does not suffice for observers to learn to suppress the location at which it appears with high probability: observers learn only if the salient object effectively distracts them, that, is, if it receives some attention. In this sense, it can be said that distractor-location suppression is learned reactively (Liesefeld et al., 2024).

However, two potential limitations of that conclusion are worth noting. First, if participants of the informative-cue group ignored our instruction to maintain fixation throughout each trial during learning, they may have shifted their gazes to the cued location and as a result, the distractor’s representation may have been too coarse to support statistical learning.^6^ However, this possibility is unlikely for two main reasons. One is that we used a bull’s eye fixation mark precisely in order to minimize involuntary eye movements (Thaler et al., 2013). The other is that our displays were small enough for the distractor’s color (which strongly differed from the majority color) to be processed even if participants did move their eyes: displays consisted of 8 items equally spaced on an imaginary circle with a radius of 2.6° of visual angle and the target location was randomly selected on each trial, such that, on average, it was distant from the high-probability distractor by less than 3° of visual angle; considering the cone distribution on the retina (e.g., Curcio et al., 1990), the high-probability distractor location was close enough to the fixated target for its color to be well discriminated.

Second, one should be cautious in claiming that statistical learning in general – rather than learned distractor-location suppression in particular – requires attention. Statistical learning is defined as encoding and using regularities in our environment (e.g., Frost et al., 2019). A sub-category that has generated much research in recent years is statistical learning as a selection-history phenomenon, that is, statistical learning by which the encoded regularities serve to *guide attention* (contextual cueing, e.g., Chun & Jiang, 1998; probability cueing, e.g., Geng & Behrmann, 2002; and learned distractor-location suppression, e.g., Ferrante et al., 2018). Here, we showed that allocating attention to the stimulus for which the regularity occurs is necessary in order to learn to guide attention away from that stimulus. Thus, it remains possible that exposure to the regularity outside the focus of attention suffices to encode it, but not for this regularity to influence attentional behavior. Accordingly, further research is needed to determine whether statistical learning that does not manifest as attentional behavior is contingent on the allocation of spatial attention to the information in which the regularity is embedded.

## Data Accessibility Statement

We commit to sharing our raw data, materials and all code on acceptance of Stage 2 manuscript.

The time-stamped pre-registered protocol, stimulus materials, data, and analysis scripts are accessible at https://doi.org/10.17605/OSF.IO/GZ6BT.

## Additional File

The additional file for this article can be found as follows:

10.5334/joc.382.s1 Supplementary materials. The planned analyses on the Inverse Efficiency Score.

## Footnotes

Turatto et al. (2018) found habituation to a distractor’s salient feature when participants did not engage in any task. As habituation to a distractor’s feature can be viewed as a strong form of statistical learning (i.e., with 100% probability for the regularity), this finding is consistent with Duncan and Theeuwes’ (2020) finding.

Due to a technical error, however, 8 participants were assigned to one location, 10 participants to another location, and 9 participants to each of the remaining two locations.

Due to a technical error, the high-probability distractor location was not fully counterbalanced across the 8 possible locations: in both groups, four locations (the cardinal locations) were assigned to 8 participants each and the remaining four locations (non-cardinal locations) were assigned to 6 participants each. However, preliminary analyses with high-probability distractor location type (cardinal vs. non-cardinal) as a between-subjects factor, confirmed that this factor was not involved in any significant effect, all *F*s < 1.

The pre-registered dependent variable was the Inverse Efficiency Score (i.e., RT/Accuracy; Townsend & Ashby, 1978). Although all our predictions were confirmed with IES as the dependent measure, inspection of the results revealed that the accuracy data did not contribute to the pattern of results: all RT results closely mirrored the IES results and there was no statistical learning for either group on accuracy. Therefore, to increase transparency and comparability with previous studies, we reported RT and accuracy results separately. The results of the pre-registered analyses on IES are reported in the Supplementary materials.

An exploratory analysis confirmed that for the informative-cue group, when trials in which the distractor location repeated from the previous trial were removed, the statistical-learning effect in the learning phase was no longer significant, *p* > .6, *d* = –.03.

We thank Dirk van Moorselaar for this comment.
