# Supplementary material for: What is the Role of Spatial Attention in Statistical Learning During Visual Search?
Source: J Cogn. 2024 Jul 11;7(1):52. doi: 10.5334/joc.382 (PMC11243762; doi:10.5334/joc.382)
Supplement: Supplementary materials. — The planned analyses on the Inverse Efficiency Score. [file joc-7-1-382-s1.pdf]

Supplementary materials

In our pre-registration of Experiment 2, we had planned to use the Inverse Efficiency Score (i.e., RT/Accuracy; IES, Townsend & Ashby, 1978) as our dependent variable. However, as the RT results tightly conformed to the IES results and none of the effects of interest reached significance on accuracy, we concluded that reporting RTs and accuracy data separately provided a more accurate description of the findings. Nevertheless, for transparency, we report the planned analyses on IES below.

Distractor interference in the learning phase

We conducted an ANOVA with distractor condition (distractor in a low-probability location vs. absent) as a within-subject variable and group (informative vs. neutral cue) as a between-subjects variable, for the learning phase.

The main effects of group and distractor condition were significant, $F(1, 110) = 214.90$, $p < .001$, $\eta_p^2 = .66$ and $F(1, 110) = 150.13$, $p < .001$, $\eta_p^2 = .58$, respectively. As predicted, the two factors interacted, $F(1, 110) = 129.52$, $p < .001$, $\eta_p^2 = .54$, indicating that distractor interference was significant for the neutral-cue group, $t(55) = 12.41$, $p < .001$, $d = 1.66$, but did not reach significance for the informative-cue group, $t(55) = 1.42$, $p = .08$, $d = .19$.

Distractor interference in the test phase

The same ANOVA for the test phase revealed significant main effects of group and distractor interference, $F(1, 110) = 23.02$, $p < .001$, $\eta_p^2 = .17$ and $F(1, 110) = 119.18$, $p < .001$, $\eta_p^2 = .52$, respectively, indicating that participants' performance was poorer in the

informative-cue group than in the neutral-cue group and when the color-singleton distractor was present in a low-probability location than when it was absent. The two-way interaction was not significant, $F < 1$, indicating that, unlike in the learning phase, the distractor interfered to the same extent during test in the informative- and in the neutral-cue groups.

Statistical learning in the learning phase

We conducted an ANOVA with statistical learning (distractor in a low-probability vs. in the high-probability location) as a within-subject variable and group (informative vs. neutral cue) as a between-subjects variable on the learning phase data.

The main effects of group and distractor condition were significant, $F(1, 110) = 230.59$, $p < .001$, $\eta_p^2 = 68$ and $F(1, 110) = 99.36$, $p < .001$, $\eta_p^2 = .48$, respectively. The interaction between the two factors was also significant, $F(1, 110) = 89.93$, $p < .001$, $\eta_p^2 = .45$, indicating that statistical learning was significant for the neutral-cue group, $t(55) = 10.10$, $p < .001$, $d = 1.35$, but not for the informative-cue group, $t < 1$, $p = .19$, $d = .12$.

Statistical learning in the test phase

We conducted the same ANOVA on the data of the test phase. The main effect of group was significant, $F(1, 110) = 20.77$, $p < .001$, $\eta_p^2 = .16$, indicating that unlike in the learning phase, participants were faster in the neutral- than in the informative-cue group. The main effect of statistical learning was not significant, $F < 1$. Crucially, the two-way interaction between the two factors was significant, $F(1, 110) = 4.02$, $p = .048$, $\eta_p^2 = .04$,indicating that statistical learning was significant for the neutral-cue group, $t(55) = 3.17$, $p = .001$, $d = .42$, but not for the informative-cue group, $t(55) = 1.01$, $p = .84$, $d = .14$.

Reference

Townsend, J. T., & Ashby, F. G. (1978). Methods of modeling capacity in simple processing systems. In J. N. J. Castellan & F. Restle (Eds.), Cognitive theory (Vol. 3, pp. 199–239). New York: Lawrence Erlbaum Associates.
